# Effects of self-myofascial release interventions with or without sliding pressures on skin temperature, range of motion and perceived well-being: a randomized control pilot trial

**DOI:** 10.1186/s13102-021-00270-8

**Published:** 2021-04-22

**Authors:** Yann Kerautret, Aymeric Guillot, Carole Eyssautier, Guillaume Gibert, Franck Di Rienzo

**Affiliations:** 1 Univ Lyon, Université Claude Bernard Lyon 1, Laboratoire Interuniversitaire de Biologie de la Motricité EA 7424, F-69622 Villeurbanne Cedex, France; 2 CAPSIX, 69450 Saint-Cyr au Mont d’Or, France; 3 Institut Universitaire de France, Paris, France

## Abstract

**Background:**

Self-myofascial release is an emerging technique in strength and conditioning. Yet, there is no consensus regarding optimal practice guidelines. Here, we investigated the acute effects of various foam rolling interventions targeting quadriceps muscles, with or without sliding pressures.

**Methods:**

We conducted a blinded randomized control pilot trial in 42 healthy weightlifting athletes over 4 weeks. Participants were randomly allocated to one of the four intervention (120 s massage routine) groups: foam rolling, roller massager, foam rolling with axial sliding pressures, foam rolling with transverse sliding pressures. Knee range of motion, skin temperature and subjective scores of the perceived heat, range of motion, muscle pain and relaxation were the dependent variables. Measurements were carried on before, after and up to 15 min (follow-up) after the massage intervention.

**Results:**

The range of motion increased immediately after the various foam rolling interventions (+ 10.72%, 95% CI 9.51 to 11.95, *p* < 0.001), but progressively returned back to the pre-intervention baseline along within the 15 min post-intervention. Foam rolling was the most effective intervention to increase skin temperature from thermographic measures (+ 14.06%, 95% CI 10.97 to 17.10, *p* < 0.001), while the increase in perceived heat was comparable in all experimental groups (107%, 95% CI 91.08 to 122.61, *p* < 0.001).

**Conclusions:**

Subjective indexes of heat, range of motion, muscle pain and relaxation improved immediately after the intervention, but also gradually returned to the pre-intervention baseline. Overall, combining foam rolling with sliding pressures did not yield additional benefits from objective measures.

**Supplementary Information:**

The online version contains supplementary material available at 10.1186/s13102-021-00270-8.

## Introduction

Self-myofascial release (SFMR) techniques, including Foam Rolling (FR), are increasingly used as part of warm-up and post-workout recovery routines in the field of strength and conditioning [1]. FR consists in back and forth rolling movements applied to specific body parts using a foam roller or a roller massager [2]. Irrespective of the massaging tool, SMFR consists in self-administering mechanical pressures to the soft tissues, typically by rolling and/or sliding movements [3–5]. Physiological structures impacted by SMFR involve the skin, the muscles and their corresponding fascia. These organs contain mechanoreceptors such as the Golgi tendon organ involved in tonus regulations, and the corpuscles of Pacini, Meissner, and Ruffini which enable proprioception by detecting subtle changes in pressures, tangential forces and fine touch [6–8]. Myofascial manipulations also impact type III and IV interstitial receptors, which have an additional role in vasodilation and pain perception [6–8].

Mechanical pressures applied to the soft tissues were associated with a wide range of positive effects. SMFR techniques elicit comparable beneficial effects to those resulting from traditional manual massages [9–11]. FR was associated with improved flexibility, range of motion and reduced pain symptoms [3, 12]. Past experiments also demonstrated its potential relevance to improve on flexibility, as assessed from range of motion measures [12, 13]. The benefits were recorded up to 20 min post-treatment, but did not appear to last beyond a 30-min threshold [14–18]. Contrary to passive stretching, the effects of FR were not associated with a corollary decline in physical performances [3]. This was shown for explosive efforts and agility measures [19–21]. It is noteworthy that the pressures exerted during FR might constitute a risk factor for vessels, nerves or bones injuries [4, 22]. Pressure exerted during FR can reach up to 516 mm/Hg [22]. This represents a five-fold increase compared to a high pressure manual massage [23]. Eliska and Eliskova demonstrated lymphatic vessel damage above a 100 mm/Hg threshold. Controlling the pressure threshold is thus crucial in FR designs [10].

No study has yet provided detailed insights on the neurophysiological factors underlying the benefits of SMFR on performance. The scientific rationale supporting the efficacy of FR interventions derives from findings in traditional manual massage [4, 11, 24]. It was postulated that SMFR could decrease tissue stiffness, break adhesions and scar tissues [25]. It was also postulated that the mechanical pressures associated with SMFR yielded sympathetic inhibition as a result of mechanoreceptors stimulation [8, 10]. This was advanced as an account to the benefits of FR on range of motion and pain symptoms, which appeared comparable to those elicited by a myofascial manipulation intervention [8, 10, 25]. It was also postulated that the mechanical pressures applied during FR on the skin, muscles and fascia would produce the necessary frictions for thixotropic effects [25]. Schleip et al. [6] advanced that energy input to the fascia through mechanical pressure or heat promoted the return of viscoelastic and thixotropic properties [3]. Past experiments measured the local skin temperature, using an infrared thermometer, a skin thermistor or thermographic cameras [24, 26–33]. While manual massage and FR yielded comparable acute positive effects on the range of motion [24, 32, 33], both techniques appeared to have a different effect on skin temperature. Immediately after a traditional manual massage, the increase in skin temperature was inconsistently found [28–31, 33]. However, no effects were observed after a FR intervention [24]. It is was postulated that manual gestures generates friction forces accounting for the rise in skin temperature. This may however be restricted to the skin and superficial layers of the muscles, and therefore might not indicate a general increase in muscle temperature comparable to that produced during warm-up. A pioneer experiment underlined temperature increases in quadriceps muscles at a depth of 2.5 cm from the skin, but not at 3.5 cm [26]. Hinds et al. [27] failed to replicate these observations, thus supporting that manual massages had negligible effect on muscle temperature.

To the best of our knowledge, only one experiment investigated whether FR impacted skin temperature. Murray et al. [24] found no effect on skin temperature, but reported positive effects on knee range of motion. Importantly, the FR tool can be used as the support with the body being mobile with the body weight being used as an external force to apply pressures. However, the body can also be the support, and the mechanical pressures being applied by manipulating the FR tool with the upper limbs. In both cases, contrary to manual massage, there is a relative absence of frictions during SFMR [32]. These sliding pressures, also qualified as shearing self-manipulations, are defined as mechanical forces that act a skin area in a parallel direction to that of the body’s surface [32]. In massage interventions including sliding pressures, the massaged limb is systematically the fixed point. The rolling mechanism of the foam roller is neutralized. Hence, massage movements with the tool trigger stretch and friction from the superficial layers of the targeted tissues. Sliding pressures rely both on the fixed point and the direction of the movement applied with the tool and imitate transverse and deep tissues stimulation, which are part of most traditional manual massage routines [34]. In the study by Gordon et al. [32], participants were asked to apply linear sliding pressures using a vibratory stick without rolls. Back and forth sliding pressures increased both the range of motion and skin temperature. The tool used in this experiment was to able generate stretch and friction forces on the soft tissues, similarly as the hands of a manual massage therapist [33, 35]. Interestingly, FR elicits an increase in stretch sensations along with range of motion improvements [3, 36]. Also, sliding pressures improved perceived relaxation and lightness [32]. Including sliding pressure strokes within FR routines might thus be associated with additional benefits.

In the present experiment, we aimed at comparing the effects of SMFR interventions with or without sliding pressures. Four independent groups received a distinct SMFR intervention targeting quadriceps muscles of the dominant leg. We hypothesized that incorporating sliding pressure movements in the FR routine would yield additional benefits on range of motion and perceived sensations associated with well-being. The range of motion was considered the primary outcome variable of this pilot trial, since it is frequently focused as the main outcome of FR interventions. We also hypothesized that including sliding pressures according to an axial or transverse direction relative to the proximal-distal axis of the muscle would increase skin temperature compared to FR without sliding pressures.

## Materials and methods

### Participants

Forty-two healthy adults (20 males, 22 females, 26.3 ± 3.6 years, 1.70 ± 0.10 m, 70.2 ± 9.8 kg, Body Mass Index: 23.6 ± 2.0) with a history of FR experience (19.2 ± 10.1 months) volunteered to participate in the experiment. Recruitment strategies consisted in poster advertising and personal contact between Crossfit® coaches and practitioners. The pilot trial inclusion criteria included regular use of FR (> 8 h per week) for the past 6 months. This implies that our sample of participants possibly had greater flexibility than the average population, due to the continuous effects of FR on this physical quality [37, 38]. Inclusion of non-naïve participants was nonetheless deemed necessary to ensure a correct execution of routines and prevent novelty bias. Exclusion criteria included the presence of any musculoskeletal, systemic, or metabolic disease that would have affected lower extremity joint range of motion, or ongoing medication with cardiovascular, pulmonary, thyroid, hyperlipidemic, hypoglycemic, hypertensive, endocrinologic, psychotropic, neuromuscular, neurological, androgenic, or anti-inflammatory implications [39, 40]. More specifically, individuals using anti-inflammatory and analgesic drugs that might affect testing, proprioception or pain perception, were excluded [36]. The present experiment was approved by the ethics committee CPP Ouest 6 (IRB 2019-A01732–55) and in accordance with the ethical standards laid down in the 1964 Declaration of Helsinki and its later amendments. All participants were banned from eating and drinking alcohol and strenuous physical activities containing caffeine and similar stimulants at least 24 h before test sessions.

### Experimental design

**Fig. 1:**
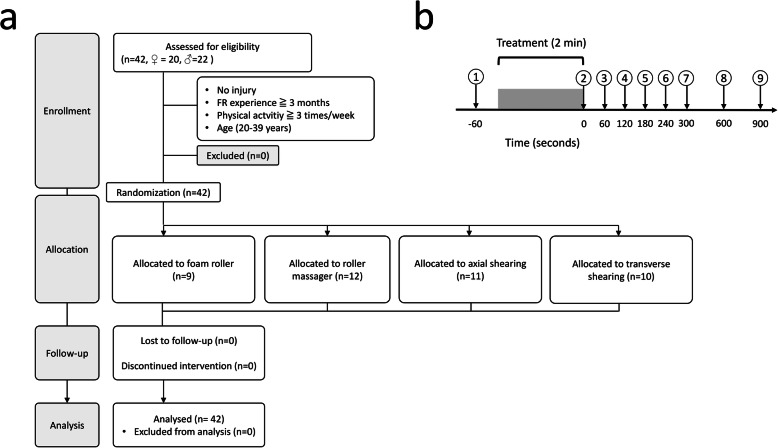
**a** Experimental design. **b** Experimental session framework depicting measurement times at baseline (5 min), and after the foam rolling interventions (up to 15 min)

The repeated measurements design was scheduled over an inclusion period of 4 weeks. The experimental design involved a familiarization session and an experimental session consisting in a pretest and an immediate posttest, followed by retention measures (Fig. 1a, b). Participants were assigned to one of the following groups: FR with a foam roller (*n* = 9, Foam rolling), FR with a roller massager (*n* = 12, Roller Massager), FR with a foam roller combined with axial sliding pressures from the top of patella to anterior superior iliac spine with a wooden stick (vertical axis) (*n* = 11, Axial sliding pressures), and FR with a foam roller combined with transverse sliding pressures (medial-lateral axis) (*n* = 10, Transverse sliding pressures) (Fig. 1a). Detailed information regarding the FR intervention across groups and the group allocation procedure are provided below. Testing was conducted between 12 A.M. and 6 P.M., and participants were instructed to refrain from any strenuous activity the day prior to testing and from taking any medication that would interfere with testing. The intervention was performed on the dominant quadriceps (leg used to kick a ball).

#### Familiarization

Participants first completed a questionnaire from which demographic information was collected, and familiarized with the FR interventions. This first visit, lasting up to 15 min, was an orientation and familiarization session to apprehend the use of the tools and techniques imposed by this experiment with instructions and a demonstration from the experimenter. In the same way, this session allowed participants to apprehend the different parameters of SMFR standardized specifically for this pilot trial; the recommended rhythm and level of pressure, the treatment time and the area to be massaged. More specifically, participants learned to change their area on the quadriceps, medial, middle and lateral parts, while simultaneously respecting the other parameters already listed. Participants were instructed to regulate their rolling so that the perceived pain would be maximum 7 on a 0–10 Numeric Rating Scale (NRS) (0: “No pain”, 10: “Worst possible pain”).

#### Foam rolling interventions

Two commercial FR tools were used across experimental conditions: a foam roller of 15*30 cm length (Blackroll, Bottighofen, Switzerland), and a roller massager of 45 cm length (Physioroom, Lancashire, England). Foam roller was a cylinder of 100% of polypropylen which was used in prior research. Roller massager was composed by a metal tube surrounded by dissociated plastic bearings. Especially for this pilot trial, a wooden massage stick was created, with a shape being like an ellipse.

In the Foam rolling group, participants were instructed to begin in a plank position and place the foam roller against the anterior face of their dominant thigh. To facilitate execution, the quadriceps was divided into two areas: i) from the top of patella to the middle of the quadriceps and ii) from the middle of the quadriceps to the anterior superior iliac spine. Participants positioned the roller above their left patella and rolled back and forth the first area four times with a cadence of one inch per second (controlled by a metronome). Participants were then instructed to stop and execute four knee flexions at 90 degrees. The same sequence was applied to the second quadriceps area. This yielded a total duration of 2 min of FR intervention. The pressure applied to the dominant quadriceps was controlled using NRS self-reports (0: “Absence of pain”; 10: “Worst possible pain”), in keeping with the methods emphasized in previous FR experiments [41]. At any time, ratings should not exceed a predetermined 7 out of 10 threshold. To control pressure, participants were allowed to place the non-massaged leg in contact with the floor and to use their hands and forearms to manage the pressure. The choice of these instructions was based on the methodology adopted in previous experiments [42–45].

In the Roller massager group, participants performed FR with a roller massager [46, 47]. They sat on the edge of a table, feet rested on a chair to place their dominant lower limb in a semi-stretched position. This position is suitable for quadriceps relaxation and to apply massaging movements with upper limbs. Participants were then instructed to roll their two quadriceps areas, as previously presented, without crossing joints. Participants followed the same instructions as the Foam rolling group (pressure level, cadence, muscle pain perception, and treatment duration). After performing the rolling movements with the roller massager, participants performed 4 knee flexions while maintaining pressure with the roller massager.

For Axial sliding pressures and Transverse sliding pressures groups, the massage routine with the foam roller was combined with either axial or transverse sliding pressures. The massage routine was the same as that described for the Foam Rolling group, but only lasted 1 min. The remaining minute was allocated to sliding pressures movements applied with a homemade wooden stick where the rolling component was neutralized. As in the Roller Massager group, the massage with the homemade wooden stick was performed sitting on a table to allow knee mobilizations. These methodological choices reproduced the methods described in experimental protocols addressing the effects of autogenous friction [48].

### Dependent variables

As psychometric indexes; we used NRS to address at the subjective level the effects of the FR intervention (Heat, Range of Motion, Muscle pain and Relaxation). As behavioral index, were recorded the flexibility of the posterior chain made from the Duncan-Ely test. As neurophysiological index, we recorded skin temperature using a thermography camera. The assessor was blinded to the group assignment but remained the same throughout the pilot trial to ensure reproducibility of the measures. No feedback was provided to participants until after completion of the design.

#### Skin temperature

**Fig. 2:**
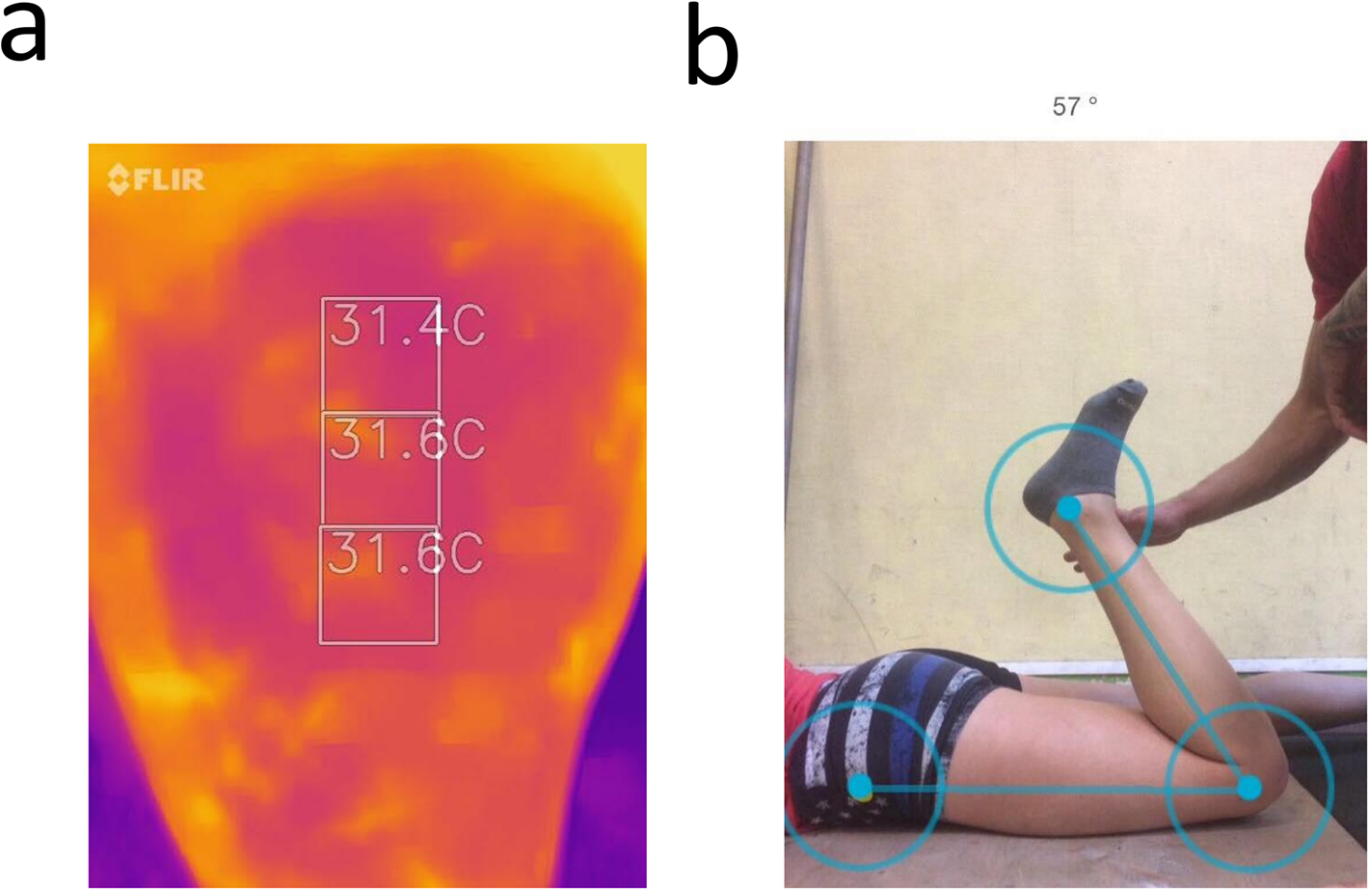
**a** Measures of skin temperature. **b** Measure of the knee range of motion

We used the FLIR-ONETM thermography camera (FLIR Systems, Frankfurt, Germany) to collect thermographic measures as participants sat on a chair. The camera yielded pictures differentiating skin temperature across a color range. The application provides skin temperature measures ranging from − 20° to 120 °C, with a sensitivity of ±0.1 °C (Fig. 2a). To ensure the reliability and reproducibility of measures, an upper rubber band was positioned on the upper-third of the quadriceps (corresponding to the center of the muscle belly). For each photo, the sensor of the camera was directed towards the rubber band, at 10 cm to optimize the resolution. In addition, a grid of three zones was used to distinguish a proximal area, a central area and a distal area. 5 min after the FR intervention, skin temperature was collected every min for 5 min. Then, skin temperature was collected every 5 min (i.e. at 10 and 15 min post-intervention).

#### Range of motion

We used a digital goniometer (CJOrtho, Collège des Jeunes Orthopédistes, Paris, France) to collect the passive knee flexion range of motion from the Duncan-Ely test [49]. The position of the camera was standardized and positioned at 200 cm of the participant. Strips of tape were placed on the floor to mark the position of the thermography camera. Passive knee range of motion recordings required a total of five repeated measurements of 15 s distributed before and after the FR intervention (Fig. 2b). Range of motion was collected immediately after the intervention, and every 5 min post-intervention (until 15 min). To complete the Duncan-Ely test, participants laid prone on a yoga mat. Their ankle was grasped by a physiological therapist who moved the knee to the end of their maximal flexion range of motion. This corresponds to the point where the knee can no longer be passively moved without providing overpressure [50]. The range of motion was collected with the optical goniometer from the video recordings of the Ducan-Ely test. The assessor controlled for compensatory movement including extern rotation leg and/or knee elevation, and lateral and/ or anterior tilt pelvis.

#### Psychometric measures

Participants were requested to provide self-reports ratings of their sensations on a 10-point NRS (0: “Not at all”, 10:“Very strong”). They were asked to rate their perceived quadriceps heat, knee range of motion, levels of pain and perceived relaxation in the quadriceps muscles. For pain ratings, the scale values were rectified for congruence purposes with the other NRS (0: “Very strong”, 10:“Not at all”). Psychometric recordings involved five repeated measures: before and after the intervention and every 5 min (until 15 min) post-intervention.

### Statistical analysis

#### Block randomization

We used R [51] and the package *blockrand* [52] to perform participants’ group assignment (block randomization). *Blockrand* generates random sequences using random block size selection, which is required to prevent randomization bias [53]. The randomization procedure was carried for in males (sample size generated: *n* = 24; 3 blocks; block sizes: *n* = 16, *n* = 4, and n = 4, respectively) and female (sample size generated: n = 24; 2 blocks; block sizes: n = 16, and *n* = 8, respectively) stratums of our sample. To grant allocation concealment, the random sequence was matched to a predetermined list of the male and female participants (i.e. corresponding to their inclusion order).

#### Data analysis

We used *nlme* [54] to run a series of linear mixed effects analyses. For all dependent variables, we first investigated the acute effect of the FR interventions [14, 32]. We built by-subject random intercept models using GROUP (Foam roller, Roller massager, Axial sliding pressures, Transverse sliding pressures) and TEST (Pretest, Posttest) as fixed effects, with interaction term. Then, we investigated *delayed* changes occurring after FR interventions. We built the random-coefficient regression models with a by-subject random intercepts using GROUP (Foam roller, Roller massager, Axial sliding pressures, Transverse sliding pressures), TIME (numeric regressor: duration in min from the Posttest, starting from 0), and PRETEST VALUE (numeric regressor: pre-intervention covariate evaluating whether participants could be regressing to different baselines) as fixed effects, with interaction terms. For range of motion and skin temperature, we also included as level-one and level-two regressor, respectively, the SUBJECTIVE RATING provided by the participants on the NRS. This was used to investigate the correlation between objective and subjective variables. For skin temperature, we included the additional fixed effect of LIMB SECTION (Proximal, Middle, Distal). For the psychometric measures, we included the fixed effect of VARIABLE TYPE (Heat, Range of motion, Muscle pain, Relaxation), to avoid replicating the analysis across all subjective dimensions (which would increase the risk for false positives). The statistical significance threshold was set up for a type 1 error rate of α = 5%. As effect sizes, we calculated partial Cohen’s f using the ad hoc procedure for linear mixed effects models implemented in the *effectsize* package [55]. Main and interactions effects were investigated post-hoc using general linear hypotheses testing of planned contrasts from the *multcomp* package. We applied Holm’s sequential corrections to control the false discovery rate [56].

#### Power/sample size considerations

Considering the pilot nature of the study, we did not run a priori power calculation or registration in a clinical trial platform. We determined the sample size based on previous studies addressing the efficacy of self-myofascial release interventions, some of which involved randomized controlled trial designs [2, 57, 58]. We accounted for the fact that randomized control trials design involving between-group comparisons usually require larger sample sizes compared to randomized crossover trials involving within-group comparisons to achieve adequate statistical power (i.e. p_1-β_ > 0.80). It is suggested that between-subject designs require 4 to 8 times more participants than within-subject designs to achieve a comparable statistical power [59]. Considering that adequate statistical power in within-subject study can be achieved using 11 participants, 42 participants was expected to constitute a reliable sample size. To further overcome statistical power limitations, we ran a posteriori power calculations for statistically significant main and interaction effects, using the *pwr* package in R [60].

## Results

Demographic information for each experimental group is provided in Table 1.

**Table 1:** Baseline characeristics (M ± SD). There were no adverse events and no subjects withdrew from the pilot trial. BMI: Body Mass Index

	Foam rolling with a foam roller	Foam rolling with a roller massager	Foam rolling with axial sliding pressures	Foam rolling with transverse sliding pressures
**Age (years)**	24.3 ± 2.4 (range 21–28)	27.2 ± 4.0 (range 24–39)	26.1 ± 2.8 (range 22–31)	27.2 ± 4.3 (range 20–35)
**Height (m)**	1.68 ± 0.1 (range 1.5–1.83)	1.75 ± 0.1 (range 1.6–1.85)	1.73 ± 0.1 (range 1.62–1.83)	1.72 ± 0.1 (range 1.62–1.83)
**Mass (kg)**	67.5 ± 11.7 (range 53.2–85.7)	73.4 ± 10.8 (range 58–95)	69.2 ± 7.5 (range 58–83)	69.4 ± 9.3 (range 55–81)
**BMI (kg/m2)**	23.7 ± 2.5 (range 19.5–27.1)	24 ± 2.4 (range 20–27.8)	23.2 ± 1.6 (range 21.4–26.4)	23.3 ± 1.4 (range 20.7–24.9)
**Foam rolling experience (month)**	21.3 ± 8.1 (range 10–36)	17.0 ± 11.3 (range 5–48)	20.2 ± 11.6 (range 5–36)	19.1 ± 9.4 (range 5–36)

### Range of motion analysis

#### Effect of the foam rolling intervention on range of motion

The backward stepwise regression model revealed that the range of motion was only affected by the main effect of TEST (F(1, 40) = 328.44; *p* < 0.001; Cohen’s f (partial) = 1.16, 95% CI [0.78, 1.53]; p_1-β_ > 0.95) and SUBJECTIVE RATING (F(1, 40) = 6.79; p < 0.001; Cohen’s f (partial) = 0.37, 95% CI [0.08, 0.66]; p_1-β_ = 0.64). Posttest range of motion was reduced compared to the Pretest (Pretest: 49.99, 95% CI [52.78, 56.37]; Posttest: 54.58, 95% CI [48.19, 51.99]; *p* < 0.001) – higher values being indicating reduced knee flexion – while SUBJECTIVE RATING of the range of motion predicted the range of motion values collected from goniometer measures (− 0.71, 95% CI [0.79, 1.23]; *p* < 0.01).

#### Time course changes in range of motion

The backward stepwise regression model revealed that the range of motion was only affected by the main effect of TIME (F(1, 125) = 266.58; *p* < 0.001; Cohen’s f (partial) = 1.46, 95% CI [1.21, 1.71]; p_1-β_ > 0.95) and PRETEST VALUES (F(1, 40) = 284.24; *p* < 0.001; Cohen’s f (partial) = 2.67, 95% CI [2.00, 3.32]; p_1-β_ > 0.95). TIME positively predicted the range of motion raw values (+ 0.28, 95% CI [0.25, 0.31]; p < 0.001). PRETEST VALUES also positively predicted the actual range of motion (+ 1.37, 95% CI [1.21, 1.53]; p < 0.001).

### Skin temperature analysis

#### Effect of the foam rolling interventions on skin temperature

**Fig. 3:**
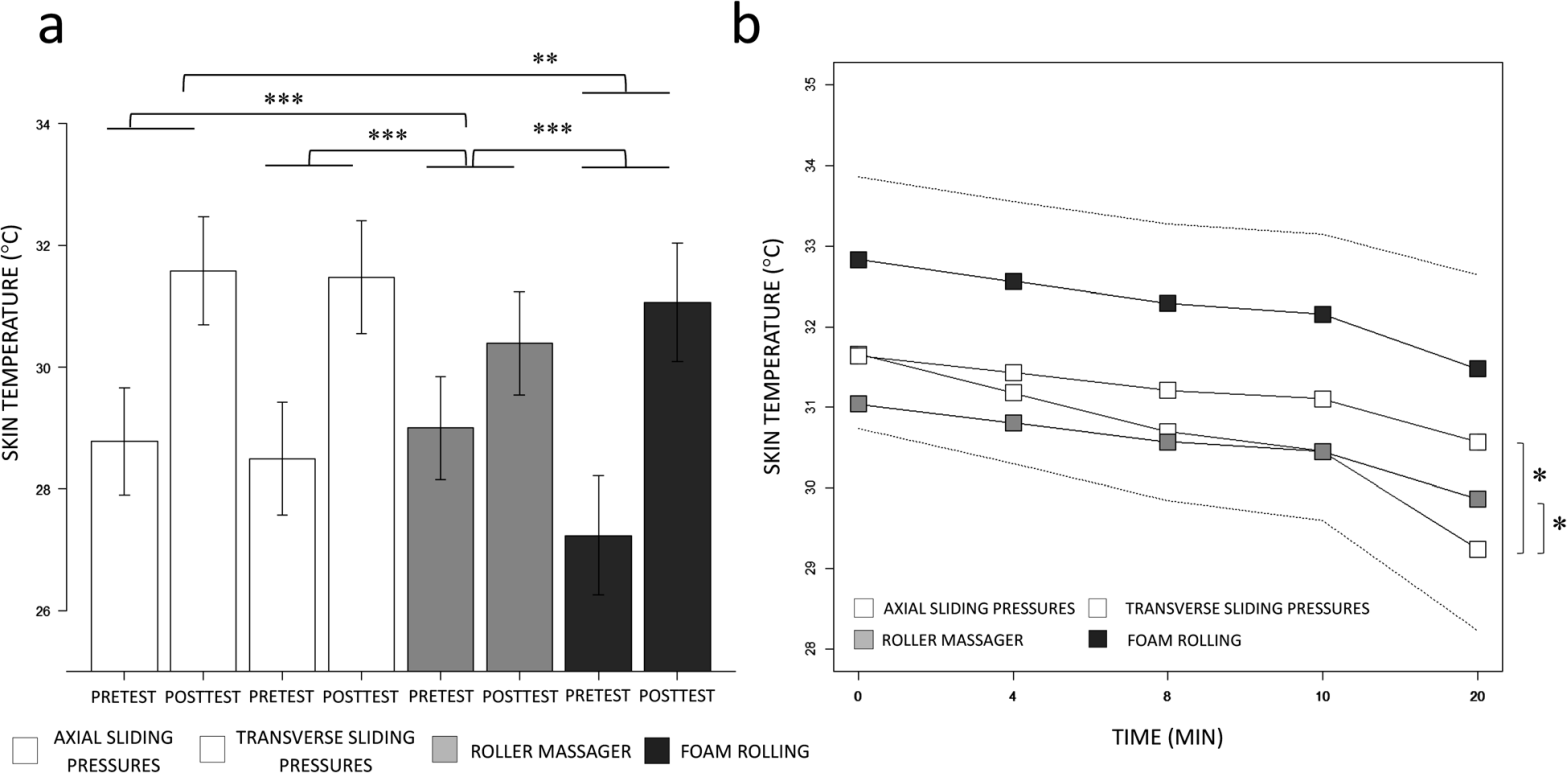
**a** Barplot display of the TEST by GROUP interaction effect on the acute effects of the foam rolling interventions on skin temperature, represented with 95% confidence intervals (error bars). **b** Regression slopes attesting the effect of TIME on skin temperature across the different classes of GROUP, presented with 95% confidence intervals (dotted lines)

The backward stepwise regression model revealed that the two-way interaction between GROUP and TEST affected skin temperature (F(1, 206) = 18.93; *p* < 0.001; Cohen’s f (partial) = 0.53, 95% CI [0.37, 0.66]; p_1-β_ = 0.81). The Pretest to Posttest variation in skin temperature during Roller massager (Pretest: 29.00, 95% CI [28.15, 29.84]; Posttest: 30.39, 95% CI [29.55, 31.23]) was reduced compared to that observed in the Axial sliding pressures group (Pretest: 28.78, 95% CI [27.90, 29.66]; Posttest: 31.58, 95% CI [30.70, 32.46]) (*p* < 0.001), Transverse sliding pressures (Pretest: 28.49, 95% CI [27.57, 29.42]; Posttest: 31.48, 95% CI [30.55, 32.40]) (p < 0.001) and Foam rolling (Pretest: 27.24, 95% CI [26.26, 29.84]; Posttest: 31.06, 95% CI [30.09, 31.23]) (p < 0.001) conditions (Fig. 3a). Also, the Pretest to Posttest variation in skin temperature during Foam rolling was superior to that recorded during Axial Sliding pressures (*p* < 0.01), while the difference between Foam rolling and Transverse sliding pressures did not reach the statistical significance threshold (*p* = 0.07) (Fig. 3a).

#### Time course changes in skin temperature

The two-way interaction between GROUP and TEST (F(3, 456) = 2.66; *p* < 0.05; Cohen’s f (partial) = 0.14, 95% CI [0.00, 0.21]; p_1-β_ = 0.75) affected skin temperature. Skin temperature decrease along with TIME was greater during Axial sliding pressures (− 0.12 °C/min, 95% CI [− 0.16, − 0.08]) compared to Transverse sliding pressures (− 0.05 °C/min, 95% CI [− 0.09, − 0.01]) (*p* < 0.05) and Roller massager (− 0.06 °C/min, 95% CI [− 0.10, 0.02]) (p < 0.05). There was no difference between Axial sliding pressures and Foam rolling (− 0.07 °C/min, 95% CI [− 0.09, 0.05]), nor between other pairwise comparisons (*p* > 0.05, Fig. 3b). We also found a main effect of TIME (F(1, 456) = 123.09; *p* < 0.001; Cohen’s f (partial) = 0.24, 95% CI [0.16, 0.32]; p_1-β_ > 0.95), SUBJECTIVE RATING (F(1, 456) = 6.16; *p* < 0.01; Cohen’s f (partial) = 0.09, 95% CI [0.00, 0.18]; p_1-β_ > 0.95) and PRETEST VALUE (F(1, 456) = 37.10; p < 0.001; Cohen’s f (partial) = 0.61, 95% CI [0.37, 0.85]; p_1-β_ > 0.95). TIME had a negative predictive relationship on skin temperature (− 0.12, 95% CI [− 0.16, − 0.08], p < 0.001). The PRETEST VALUE in skin temperature and SUBJECTIVE RATINGS of skin temperature, both used as covariates, had a positive predictive relationship (+ 1.05 °C, 95% CI [0.68, 1.42], *p* < 0.001; + 0.24 °C, 95% CI [0.01, 0.48], *p* < 0.05).

### Psychometric analysis

#### Effect of the foam rolling interventions on subjective scores

The backward stepwise model selection revealed that the two-way interaction between TEST and VARIABLE TYPE (F(3,287) = 84.74; *p* < 0.001; Cohen’s f (partial) = 0.82, 95% CI [0.68, 0.95]; p_1-β_ > 0.95) affected the subjective scores. The difference in perceived Heat between the Pretest and the Posttest (Pretest: 3.14, 95% CI [2.74, 3.54], Posttest: 6.50, 95% CI [6.09, 6.90]) was greater than the difference in perceived Pain between the Pretest and the Posttest (Pretest: 8.79, 95% CI [8.38, 9.19], Posttest: 7.09, 95% CI [6.96, 7.50]) (*p* < 0.001), the difference in perceived Relaxation between the Pretest and the Posttest (Pretest: 5.07, 95% CI [4.66, 5.47], Posttest: 6.33, 95% CI [5.93, 6.73]) (p < 0.001), and the difference in perceived Range of motion between the Pretest and the Posttest (Pretest: 4.90, 95% CI [4.50, 5.30], Posttest: 6.83, 95% CI [6.43, 7.23]) (p < 0.001). Pain scores decreased from the Pretest to the Posttest, hence exhibited a distinct profile than the increase recorded in subjective ratings of Relaxation (*p* < 0.001) and Range of motion (p < 0.001).

#### Time-course changes in subjective scores

**Fig. 4:**
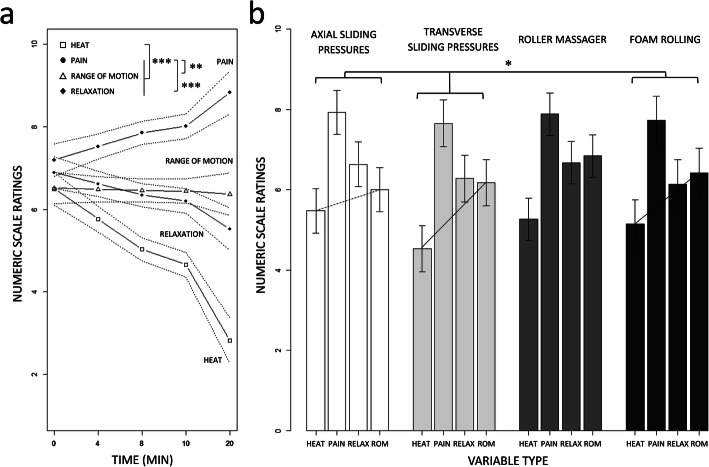
**a** Regression slopes attesting of the TIME by GROUP interaction effect on subjective ratings of sensations associated with well-being (post-intervention). Average values are presented with 95% confidence intervals (dotted bars). **b** Barplot display illustrating the GROUP by VARIABLE TYPE interaction post-intervention. Average values are presented with 95% confidence intervals (dotted bars)

The backward stepwise model selection revealed that the two-way interactions between TIME and VARIABLE TYPE (F(3,610) = 32.93, *p* < 0.001, Cohen’s f (partial) = 0.48, 95% CI [0.39, 0.56]; p_1-β_ > 0.95) affected the subjective ratings. The interaction between GROUP and VARIABLE_TYPE also reached the statistical significance threshold (F(9,610) = 1.80, *p* = 0.05, Cohen’s f (partial) = 0.16, 95% CI [0.00, 0.23]; p_1-β_ = 0.20). The negative influence of TIME on Heat scores (− 0.10/min, 95% CI [− 0.14, − 0.06]) was greater than that recorded for Relaxation (− 0.01/min ± 0.02, 95% CI [− 0.05, 0.03]), Pain (+ 0.08/min, 95% CI [0.04, 0.12]) and Range of motion (− 0.12/min ± 0.03, 95% CI [− 0.18, − 0.06]) (all *p* < 0.001) scores (Fig. 4a). Also, the influence of TIME on Pain ratings was superior to that for Relaxation *p* < 0.01) and Range of motion (p < 0.001), both of which were negative (Fig. 4a). Irrespective of TIME, the difference between Heat and Range of motion subjective scores in the Axial sliding pressures group (Heat: 5.47, 95% CI [4.91, 6.83]; Range of motion: 6.00, 95% CI [5.44, 6.53]) was reduced compared to that recorded in both the Transverse sliding pressures (Heat: 4.52, 95% CI [3.94, 5.11]; Range of motion: 6.17, 95% CI [5.58, 6.76]) and Roller massager (Heat: 5.27, 95% CI [4.74, 5.81]; Range of motion: 6.85, 95% CI [6.32, 7.39]) groups (both *p* < 0.05, Fig. 4b).

## Discussion

### Pilot trial outcomes

The primary aim of the pilot trial was to investigate whether sliding pressures yielded additional benefits compared to conventional FR interventions on skin temperature, range of motion and perceived sensations. Contrary to our initial hypothesis, combining sliding pressures to FR did not appear to elicit additional benefits. Conventional FR without sliding pressures elicited a greater increase in skin temperature, while FR with the roller massager had a more limited effect. The skin temperature increase after FR was associated with higher perceived heat post-intervention compared to axial sliding pressures. Also, despite a comparable increase in skin temperature, skin temperature decrease post-intervention was faster after axial sliding pressures compared to transverse sliding pressures. All SMFR interventions improved knee range of motion. The acute and time-course effects of SMFR on knee range of motion and skin temperature were overall associated with corollary changes in the perceived sensations.

### Acute effects of FR interventions

We expected differences in range of motion improvements due to the adjunction of sliding pressures to the FR routine. However, all SMFR interventions yielded comparable acute effects. Since measurements were conducted at the single session level in experienced athletes, we cannot rule out ceiling effect. The results nonetheless challenge our initial hypothesis. Regardless the experimental condition, 5.85° of range of motion improvement was recorded between the pre- and the post-test. The overall beneficial effects across all FR interventions is consistent with literature findings [44, 45]. Several hypotheses were advanced as an account to range of motion increases after a short bout of FR (with or without sliding pressures). Heat and pressures may alter thixotropic and viscoelastic properties of the fascia [3, 6, 25]. Rivera et al. [61] underlined that FR might elicit an increase in local blood flow that can facilitate range of motion due to a local increase in muscle temperature [32]. Also, the effect of heat and pressure on proprioceptive organs might downregulate the muscle tone through the activation of interstitial receptors type III, IV, and Ruffini corpuscles [6, 8, 25]. SMFR interventions might thus retroact on the autonomic nervous system and balance sympathetic and parasympathetic outputs to the muscles [6, 8, 25]. An effect of pressures on pain modulatory systems through the activation of interstitial nociceptive organs was also postulated [6, 25]. These assumptions enable to contextualize present findings within a psychophysics (rather than biomechanical) framework.

There is recent evidence of skin temperature increases as a result of FR or sliding pressures [32, 61]. Conversely, some studies did not report such effects [24]. It is noteworthy that past experiments did not compare the effects of FR with or without sliding pressures in a single design. Here, all FR interventions elicited an acute increase in skin temperature, which was however greater after the FR intervention *without* sliding pressures. This may account for an greater increase in local blood flow as a result of the FR pressures compared to sliding pressures – which remain superficial [27, 28, 30, 32, 61]. The hypothesis of causal relationship between skin temperature and local blood flow increases is not new [31, 33]. Our initial hypothesis was that the adjunction of axial or transverse sliding pressures to FR would elicit greater increases in skin temperature. Unexpectedly, both sliding pressures groups exhibited comparable increases in skin temperature, both of which did not outperform that observed in the FR group. We therefore suggest that pressures, rather than frictions, are likely to affect the local blood flow.

Psychometric data demonstrated a beneficial effect of FR on perceived heat, range of motion and relaxation. Perceived pain also increased, but remained in the lower range of the NRS. The perceived sensation associated with well-being which exhibited the greatest increase from the pre- to the posttest, irrespective of the FR intervention, is heat. This is congruent with the increase in skin temperature observed from thermographic measures. To explain the general increase in subjective reports of perceived heat, we hypothesize that the heat generated by FR techniques stimulated heat receptors. The presence of thermoreceptors in the dermis could explain the increase in subjective reports of both perceived heat and relaxation [62, 63]. For the subjective reports of perceived range of motion, we hypothesize that the pressures exerted on soft tissues and fascia might trigger mechanoreceptors and interstitial type III and IV afferents that modulate sympathetic/parasympathetic activation [6, 8, 25]. The mechanical pressures could also induce noxious inhibitory control (DNIC), facilitating habituation to discomfort [64]. This would increase in tolerance to the pain associated with the stretch endpoint could help to achieve a higher range of motion [36]. While this remains hypothetical, the combined effect of FR interventions on cutaneous heat and noxious inhibition would increase subjective reports of perceived relaxation. Overall, the roller massager and the wooden stick massage tools achieved sufficient pressure to elicit these effects.

### Time-course change in skin temperature, range of motion and perceived sensations after FR interventions

An original approach in the present design is the quantification of time-course changes in the dependent variables after FR interventions. All dependent variables regressed back to baseline during the minutes following the intervention [65]. Yet, the repeated practice of FR interventions could result in more permanent improvements, as shown in several FR experiments [38, 47]. From a strictly practical standpoint, however, coaches and athletes seeking acute effects should ensure that the end of the warm-up including FR, and the beginning of the effort, remains short. This was particularly the case for the range of motion, which exhibited a regular decrease (+ 0.28°/min in knee range of motion), comparable across experimental conditions. The skin temperature decreased by 0.18 °C per minute overall, but was more pronounced in the axial sliding pressures group compared to both transverse sliding pressures and roller massager groups. This could indicate that the massage intervention had a more superficial effect on the tissues. The linear profile of temperature decline in the axial sliding pressures group was comparable to that recorded in the FR group, but the baseline was also higher in the latter due to a more pronounced acute effects of the massage intervention on skin temperature. Likewise, the subjective ratings of perceived sensations declined by 0.4 point per minute on the NRS overall. However, perceived pain tended to decrease, possibly indicating that minor discomfort elicited by the pressures dissipated during the minutes post-intervention. Perceived heat ratings declined more rapidly than those of perceived relaxation and range of motion. This is of practical relevance for athletes seeking for improved sensations through FR interventions as part of their warm-ups. Eventually, the overall ratings of heat post-intervention (irrespective of the time elapsed in minutes) after axial sliding pressures appeared higher than that recorded after transverse sliding pressures and FR, since it remained within the range of perceived range of motion. Indirectly, this could argue in support of a greater efficacy of axial sliding pressures compared to transverse sliding pressures with regards to the increase the perceived heat, specifically with regards to the maintenance this effect during minutes following the intervention. However, these effects remain subjective, and were not corroborated by the profile obtained from objective measures.

### Implications and future directions

The present findings confirm the usefulness of FR techniques to improve range of motion [32, 44, 45]. The transient nature of the acute effects might account for the absence of structural change, e.g. microarchitecture of cell cytoskeleton or mechanical properties of the myofilaments, at the single-session level. Present findings, nonetheless, have meaningful practical implications for both physiotherapists and coaches. As emphasized in several experiments, FR interventions with or without sliding pressures may be appropriate as part of warm-up routines [1, 3–5]. Warm-up is defined as a period of preparatory exercise and help to minimize the risk of injury [66]. In strength and conditioning, several stages are identified in warm-up routine frameworks. First, athletes perform mobility and stability exercises, i.e. referred to as “*pillar preparation*” [67]. This stage facilitates forthcoming performances and plays a role in injury prevention [68]. Then, warm-up includes a general and a specific stage. The general warm-up consists of simple and varied body weight movements, while specific warm-up consists in competition gestures and is frequently referred to as specific heating. The intensity increment during the warm-up can yield preliminary fatigue and, in some situations, injuries [69]. Since the benefits of present FR interventions were limited in time and yielded rather comparable benefits, our results advocate for their use before any type of physical exertion. Range of motion improvements could increase motor efficiency and potentiate the effects of both competition and training performances. An increase in the local muscle temperature facilitates performance through reduced stiffness, improved nerve-conduction, strength-velocity profiles, energy supply and thermoregulatory strain [70]. However, changes must occur both at the superficial and deep layers of the soft tissues to positively impact training and performance [70]. Here, the group which exhibited higher increases in skin temperature did not achieve higher range of motion. Sliding pressures, axial or transverse, did not seem to yield additional benefits. It is possible that the rise in temperature was restricted to the superficial layers and/or insufficient to have a predictive value on objective measures of performance. This is particularly relevant considering that the effects of a full warm-up intervention cannot be restricted to local effects on temperature, but also involve among several physiological factors improved blood delivery and utilization in the tissues [70, 71]. Second, the range of motion is not only determined by changes in muscle temperature. Mechanical pressure produced by FR tools may act upon pain perception through stimulation of afferent central nociceptive pathways and descending anti-nociceptive pathways, i.e., diffuse noxious inhibitory control [1].

If the purpose is to improve athletes’ sensations of well-being, FR interventions with sliding pressures may be considered for their potential to impact subjective ratings. This information may be particularly relevant to athletes seeking to improve their feelings and confidence during competitive events. This is relevant considering that the warm-up also represents an important component of the psychological preparation to a competitive event [66, 72]. The improvements observed after FR on perceived sensations (relaxation, range of motion and heat) thus provides further scientific rationale for their use as part of warm-up. The results showed that perceived heat was the sensation associated with well-being which was the most positively impacted by the intervention. Improvements in perceived heat appeared more easily maintained after axial sliding pressures. These short-term changes in perceived sensations could be a powerful asset for athletes during the last minutes preceding a competing event.

While the effects were not maintained in the absence of practice with a return to the pre-intervention baseline during the 15 min following the intervention, longer term benefits may be expected with the regular practice of FR interventions on range of motion and indexes of well-being. Future investigations might therefore address the relevance of repeatability and trainability factors in the framework of axial and transverse sliding pressures self-manipulation.

### Limitations

A main limitation of this pilot trial may be the lack of a non-intervention group. However, there is compelling evidence that no change in the main dependent variables targeted by self-massage intervention occur in the absence of treatment intervention [44]. Future research should establish in greater details whether skin temperature is a relevant marker to investigate the efficacy of manual massage and FR techniques, specifically with regards to the effects on range of motion and performance outcomes. Apart from skin temperature, all dependent variables were comparably affected by the different foam rolling interventions. Second, we did not implement objective monitoring of the pressures exerted by athletes on their soft tissues when performing FR with the foam roller, the roller massager or wooden stick. Some authors used a force platform to index pressures applied using foam rolling (with the foam roller on the floor) relative to the individual body weight, but could not quantify the contact surface indicative of pressures relative a given pressure area [24, 73]. Such methods would not be applicable to SFMR with a roller massager or wooden stick. To overcome this limitation, some researchers used a custom device controlling for force and speed steadiness [46, 57]. However, such rolling-apparatus requires the intervention of third party, which would not provide the ecological validity with regards to how athletes implement self-myofascial release/foam rolling as part of their training routines [74]. Despite our standardization precautions, we suspect that the foam roller caused more pressure than the two other tools, positively impacting skin temperature without concomitant improvement in range of motion. The pressure exerted with the upper limbs in the case of FR interventions involving sliding pressure was possibly reduced. Yet, this limitation is inherent to the methods which involve distinct technical components. It might also be objected that the intensity of the pressure may not be a crucial factor mediating the effects on performance, particularly with regards to the range of motion, since similar gains are obtained across a variety of foam roller densities [44].

## Conclusion

The present investigation compared four FR interventions. Conventional FR emerged as the most effective method to elicit acute improvements in skin temperature while concomitantly improving the range of motion. Methods combining classical FR and sliding pressures did not yield additional acute benefits. Each yielded a comparable pattern of results, hence offering a wide range of alternatives to design efficient warm-up interventions. Although these methods are not enough to replace dynamic warm-up exercises to increase body temperature in depth, they overall represent an original and novel approach to the use of FR. Sliding pressures is an uncommon method, but has the advantage of allowing greater control over the level of pressure exerted on the soft tissues. Indeed, as with the roller massager, wooden stick massages are convenient for controlling the pressure applied to the quadriceps and control the sites of pressure due to manipulation of the massaging tool with the upper limb. Strength and conditioning coaches and physiotherapists may therefore find value in this tool when designing their interventions. Future investigations might address the specific relevance and specificities of FR interventions with sliding pressures in clinical populations.

## Supplementary Information

**Additional file 1.**

## Data Availability

The datasets used and/or analyzed during the current pilot trial are available from the corresponding author on reasonable request.
