# Supplementary material for: Effects of self-myofascial release interventions with or without sliding pressures on skin temperature, range of motion and perceived well-being: a randomized control pilot trial
Source: BMC Sports Sci Med Rehabil. 2021 Apr 22;13:43. doi: 10.1186/s13102-021-00270-8 (PMC8063437; doi:10.1186/s13102-021-00270-8)
Supplement: Supplementary file 1 — Additional file 1. [file 13102_2021_270_MOESM1_ESM.docx]

Assessed for eligibility (n= 42)

Randomized (n= 42)

## Enrollment

Screened prior to eligibility assessment (n= 42)

Excluded (n= 0)

♦  Reasons (n= 0)

## Screened

## Allocation

Assessed for objective 1 (n= 10)

Assessed for objective 2 (n= 10), Etc ...

Etc ..

Assessed for objective 1 (n= 11)

Assessed for objective 2 (n= 11), Etc ...

Etc ..

Assessed for objective 1 (n= 12)

Assessed for objective 2 (n= 13), Etc ...

Etc ..

Lost to follow-up (give reasons) (n= 0)

Discontinued intervention

(give reasons) (n= 0)

Lost to follow-up (give reasons) (n= 0)

Discontinued intervention

(give reasons) (n= 0)

Lost to follow-up (give reasons) (n= 0)

Discontinued intervention

(give reasons) (n= 0)

## Assessment

Assessed for objective 1 (n= 9)

Assessed for objective 2 (n= 9), Etc ...

Etc ..

Lost to follow-up (give reasons) (n= 0)

Discontinued intervention

(give reasons) (n= 0)

## Follow-Up

Allocated to foam roller intervention

(n= 9)

♦ Received allocated intervention (n= 9)

♦ Did not receive allocated intervention (give reasons) (n= 0)

Allocated to roller massager intervention

(n= 12)

♦ Received allocated intervention (n= 12)

♦ Did not receive allocated intervention (give reasons) (n= 0)

Allocated to transverse sliding intervention

(n= 10)

♦ Received allocated intervention (n= 10)

♦ Did not receive allocated intervention

(give reasons) (n= 0)

Allocated to axial sliding intervention

(n= 11)

♦ Received allocated intervention (n= 11)

♦ Did not receive allocated intervention (give reasons) (n= 0)

Excluded (n= 0)

♦  Not meeting inclusion criteria (n= 0)

♦  Declined to participate (n= 0)

♦  Other reasons (n= 0)
